# Age-dependent pattern of cerebellar susceptibility to bilirubin neurotoxicity *in vivo* in mice

**DOI:** 10.1242/dmm.016535

**Published:** 2014-07-25

**Authors:** Giulia Bortolussi, Gabriele Baj, Simone Vodret, Giulia Viviani, Tamara Bittolo, Andrés F. Muro

**Affiliations:** 1 International Centre for Genetic Engineering and Biotechnology (ICGEB), 34149 Trieste, Italy; 2 Basic Research and Integrative Neuroscience (BRAIN) Centre for Neuroscience, Department of Life Sciences, University of Trieste, 34127 Trieste, Italy

**Keywords:** Neonatal jaundice, *Ugt1*, Phototherapy, BIND, Mouse model

## Abstract

Neonatal jaundice is caused by high levels of unconjugated bilirubin. It is usually a temporary condition caused by delayed induction of UGT1A1, which conjugates bilirubin in the liver. To reduce bilirubin levels, affected babies are exposed to phototherapy (PT), which converts toxic bilirubin into water-soluble photoisomers that are readily excreted out. However, in some cases uncontrolled hyperbilirubinemia leads to neurotoxicity. To study the mechanisms of bilirubin-induced neurological damage (BIND) *in vivo*, we generated a mouse model lacking the Ugt1a1 protein and, consequently, mutant mice developed jaundice as early as 36 hours after birth. The mutation was transferred into two genetic backgrounds (C57BL/6 and FVB/NJ). We exposed mutant mice to PT for different periods and analyzed the resulting phenotypes from the molecular, histological and behavioral points of view. Severity of BIND was associated with genetic background, with 50% survival of C57BL/6‑*Ugt1*^−/−^ mutant mice at postnatal day 5 (P5), and of FVB/NJ-*Ugt1*^−/−^ mice at P11. Life-long exposure to PT prevented cerebellar architecture alterations and rescued neuronal damage in FVB/NJ-*Ugt1*^−/−^ but not in C57BL/6-*Ugt1*^−/−^ mice. Survival of FVB/NJ-*Ugt1*^−/−^ mice was directly related to the extent of PT treatment. PT treatment of FVB/NJ-*Ugt1*^−/−^ mice from P0 to P8 did not prevent bilirubin-induced reduction in dendritic arborization and spine density of Purkinje cells. Moreover, PT treatment from P8 to P20 did not rescue BIND accumulated up to P8. However, PT treatment administered in the time-window P0–P15 was sufficient to obtain full rescue of cerebellar damage and motor impairment in FVB/NJ-*Ugt1*^−/−^ mice. The possibility to modulate the severity of the phenotype by PT makes FVB/NJ-*Ugt1*^−/−^ mice an excellent and versatile model to study bilirubin neurotoxicity, the role of modifier genes, alternative therapies and cerebellar development during high bilirubin conditions.

## INTRODUCTION

Neonatal jaundice occurs in more than 60% of normal newborns during their first week of life (Bhutani and Johnson, 2009a; Bhutani and Johnson, 2009b; Karen et al., 2009) as a result of excessive unconjugated bilirubin (UCB) formation and transient inability of the neonatal liver to clear bilirubin rapidly enough from the blood owing to delayed expression of *UGT1A1*, encoding UDP-glucuronosyltransferase 1a1 (UGT1A1). In fact, the key event in bilirubin detoxification is the glucuronidation reaction, which is accomplished by UGT1A1 in the liver (Bosma et al., 1994). Severe jaundice is also the hallmark of Crigler-Najjar syndrome type I (CNSI), in which mutations in the *UGT1A1* gene result in the complete and life-long inactivity of the UGT1A1 enzyme.

Modest elevations of plasma UCB in newborns and adults are considered beneficial owing to the antioxidant properties of bilirubin (Doré and Snyder, 1999; Doré et al., 1999). The work on Gilbert’s syndrome increasingly demonstrates the long-term beneficial effects of low UCB plasma concentrations in averting numerous adult maladies, including risk for certain cancers and atherosclerosis (Bulmer et al., 2013; Erlinger et al., 2014; Horsfall et al., 2012; Novotny and Vítek, 2003; Schwertner and Vítek, 2008; Vítek et al., 2002). However, severe hyperbilirubinemia is toxic to the developing central nervous system (Ostrow et al., 2004; Ostrow et al., 2003). Prolonged and uncontrolled high levels of UCB lead to bilirubin encephalopathy (BE) and subsequently kernicterus. In developing countries, up to 35% of newborns with kernicterus die, and some of the survivors suffer from permanent neurological sequelae (Kaplan et al., 2011).

Bilirubin targets specific brain regions, such as the basal ganglia, cochlear, oculomotor nuclei and cerebellum (Lauer and Spector, 2011; Watchko, 2006b). The spectrum of neurological deficits is severe and can include movement disorders such as athetoid dystonic cerebral palsy and oculomotor palsies, sensorineural deafness, auditory dysfunctions, and dental dysplasia. Despite the severely disabling cerebral palsy and hearing deficits, most individuals suffering from bilirubin encephalopathy have normal cognitive capacities. There is evidence that even moderate neonatal jaundice can result in subtle neuronal damage, with delayed motor development and minimally impaired cognitive functions that are unapparent during the neonatal period but lead to development or neurological impairment that becomes evident later in life (Chen and Kang, 1995; Oh et al., 2003).

High levels of UCB are conventionally treated by phototherapy (PT). Light energy (emission range 400–525 nm, peak emission 450–460 nm) is absorbed by UCB as it circulates in skin capillaries, resulting in the conversion of insoluble bilirubin into water-soluble photoisomers that are eliminated into the bile without the need of liver conjugation (Maisels and McDonagh, 2008). PT is generally very effective in preventing transient hyperbilirubinemia in healthy neonates, because the hepatic conjugation system rapidly matures. However, in some cases high UCB levels concomitant with other risk factors, such as prematurity, hemolysis, sepsis, dehydration, early hospital discharge or lack of family training towards jaundice, can lead to serious neurological outcomes and ultimately death (Kaplan and Hammerman, 2005; Watchko and Tiribelli, 2013). In addition, individuals with CNSI, who need lifelong PT treatment for up to 14 hours per day, respond temporarily to PT and are at constant risk of developing brain damage unless liver transplantation is performed (Fagiuoli et al., 2013).

**TRANSLATIONAL IMPACT**

**Clinical issue**

Neonatal jaundice is caused by high serum levels of unconjugated bilirubin, a yellow pigment that is the breakdown product of hemoglobin. It is more common and severe in preterm than in term babies, and is usually a temporary condition caused by delayed induction of UDP-glucuronosyltransferase 1a1 (Ugt1a1), which conjugates bilirubin for excretion. The condition can be treated with phototherapy, which converts bilirubin into a water-soluble form. Left untreated, severe neonatal hyperbilirubinemia, which is also the hallmark of Crigler-Najjar syndrome type I (a genetic disorder caused by mutations in the *UGT1A1* gene), can damage the nervous system, leading first to bilirubin encephalopathy and then to kernicterus (‘yellow’ staining of the brain tissue due to accumulation of bilirubin). About 70% of newborns who develop kernicterus die within a few days and survivors often have permanent neurological deficits. However, the extent of neurological damage caused by similar levels of unconjugated bilirubin varies widely, which makes it hard to assess the risk threshold of hyperbilirubinemia or to develop intervention guidelines.

**Results**

The mechanism of bilirubin neurotoxicity remains poorly understood because of the limitations of existing cellular and animal models. In this study, the authors developed a mouse model bearing a null mutation in the *Ugt1* gene that results in early neonatal lethality due to hyperbilirubinemia. They investigated bilirubin neurotoxicity in two genetic backgrounds and showed that susceptibility to bilirubin damage and the survival of mutant animals is strain-specific, a result that underscores the importance of modifier genes in the modulation of bilirubin toxicity. Using phototherapy at different times after birth, the authors identified the critical window of neuronal susceptibility to bilirubin toxicity and showed that the neurotoxic effects of bilirubin depend on the developmental stage of the cerebellum. Finally, they reported that phototherapy from birth to day 15 is sufficient to rescue mutant mice from cerebellar damage and motor impairment.

**Implications and future directions**

The results obtained in the mouse model developed here closely mirror the effects of hyperbilirubinemia in preterm infants. Thus, this new model will help the characterization of the molecular mechanisms of bilirubin toxicity during this critical period of human development. Moreover, the ability to modulate the phenotype severity by applying phototherapy for different periods makes this model a valuable and versatile setting in which to study bilirubin toxicity at different developmental stages, the effects of modifier genes, and the effectiveness of new therapeutic approaches.

With the aim of studying the mechanisms of bilirubin toxicity *in vivo*, we developed a mouse model bearing a null mutation in the *Ugt1* gene, resulting in early neonatal lethality due to bilirubin toxicity (Bortolussi et al., 2012). These mutant animals develop severe jaundice soon after birth and die within 11 days, showing significant cerebellar alterations and the major features of severe neonatal jaundice (Bortolussi et al., 2012). We previously showed that PT treatment of these mutant mice extended their median survival from 5 to 19 days, but it was not sufficient to rescue their lethal phenotype (Bortolussi et al., 2012).

In the present work we transferred the *Ugt1* genetic mutation into the FVB/NJ and C57BL/6 mouse strains, and analyzed the neurodevelopmental effects of bilirubin toxicity in these lethal murine models of neonatal jaundice. We showed that susceptibility to bilirubin damage and, consequently, survival of mutant animals is strain-specific, with a significant increase in the survival of FVB/NJ-*Ugt1*^−/−^ compared with C57BL/6-*Ugt1*^−/−^ mice, the cerebellum being the most affected organ in both mutant strains. Cerebellar susceptibility to bilirubin resulted in the alteration of its architecture, with the important degeneration of Purkinje cell (PC) dendritic arbor, which was prevented by age-dependent PT treatment. We identified the critical window of neuronal susceptibility to bilirubin toxicity and analyzed how high bilirubin levels affect brain development during the neonatal period. We showed that FVB/NJ-*Ugt1*^−/−^ mutant mice were fully rescued when PT treatment was administered from birth to 15 days of age.

One of the advantages of the presented model resides in the possibility to modulate phenotype severity by applying PT for different periods, allowing the study of physiological and biochemical implications of bilirubin toxicity at the desired developmental stage.

## RESULTS

### Phenotype severity is associated with the genetic background

**Fig. 1.**
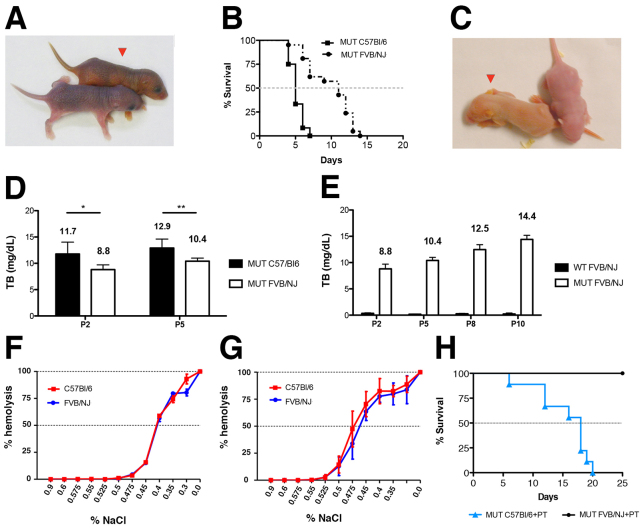
**Comparison between C57BL/6-*Ugt1*^−/−^ and FVB/NJ-*Ugt1*^−/−^ mice.** (A) Appearance of a C57BL/6-*Ugt1*^−/−^ mouse (red arrowhead) and its WT littermate at P2. (B) Kaplan-Meier survival curves of C57BL/6-*Ugt1*^−/−^ (black line with ■, *n*=12) and FVB/NJ-*Ugt1*^−/−^ mice (dashed black line with ●, *n*=21) under normal light conditions (*P*≤0.001). (C) Appearance of an FVB/NJ-*Ugt1*^−/−^ mouse (red arrowhead) and its WT littermate at P2. (D) Total plasma bilirubin levels in C57BL/6-*Ugt1*^−/−^ and FVB/NJ-*Ugt1*^−/−^ mice. Values represent means±s.d. (mg/dl). Two-way ANOVA, *P≤0.05 and **P≤0.01, C57BL/6-*Ugt1*^−/−^ versus FVB/NJ-*Ugt1*^−/−^ at P2 and P5, respectively. (E) Time course of total plasma bilirubin levels in FVB/NJ *Ugt1*^+/+^ (WT) and FVB/NJ *Ugt1*^−/−^ (MUT) mice. Two-way ANOVA. Interaction time-genotype, *P*≤0.0001; time, *P*≤0.0001, genotype, *P*≤0.0001. Values represent means±s.d. (mg/dl). (F) Osmotic fragility (OF) test of erythrocytes from P5 WT C57BL/6 and FVB/NJ animals. (G) OF test of erythrocytes from adult WT C57BL/6 and FVB/NJ animals. Results are expressed as percentage of lysis in graded salt concentrations (mean±s.d. of five males and five females). C_50_ values were determined by logarithmic linearization of the OF curve. (H) Kaplan-Meier survival curves of C57BL/6-*Ugt1*^−/−^ (blue line with ▲, *n*=10) and FVB/NJ-*Ugt1*^−/−^ (black line with ●, *n*=10) mice under PT treatment (*P*≤0.001).

We have previously reported the generation of a mouse model of neonatal severe hyperbilirubinemia bearing a targeted mutation in the *Ugt1* gene, which resembles the human CNSI from both the genetic and phenotypic points of view (Bortolussi et al., 2012). To reduce variability in the phenotype analysis, a more defined genetic background was obtained by backcrossing *Ugt1* mutant mice for more than nine generations to wild-type (WT) C57BL/6 mice, reaching at least 99.8% of C57BL/6 genetic background. Owing to the lack of Ugt1a1 activity, homozygous mutant mice developed hyperbilirubinemia within 36 hours after birth, as evident by the yellow staining of their skin (Fig. 1A), as already reported (Bortolussi et al., 2012). In this more defined genetic background, we observed a steeper shape of the mortality curve than found previously (Bortolussi et al., 2012), with 50% mortality at postnatal day 5 (P5) (Fig. 1B). To study the effects of *Ugt1* deficiency in another genetic background, we transferred the mutation into the FVB/NJ strain, reaching at least 99.8% of FVB/NJ genetic background.

As in the C57BL/6 background, FVB/NJ-*Ugt1*^−/−^ mice were visibly jaundiced as early as 36 hours after birth (Fig. 1C). Interestingly, FVB/NJ-*Ugt1*^−/−^ mice showed a less severe phenotype than C57BL/6-*Ugt1*^−/−^ mice, with 50% survival at 11 days after birth (P11; Fig. 1B). Prior to death, mutant mice showed features of bilirubin encephalopathy such as lethargy, dystonia and seizures (supplementary material Movie 1).

Determination of plasma total bilirubin (TB) showed that levels in C57BL/6-*Ugt1*^−/−^ mice were significantly higher than in FVB/NJ-*Ugt1*^−/−^ mice at both time points analyzed (Fig. 1D; two-way ANOVA, interaction time-genotype, NS; time, *P*≤0.05, genotype, *P*≤0.0005; Bonferroni’s post-hoc comparison test: *P*≤0.05 and *P*≤0.01 C57BL/6-*Ugt1*^−/−^ versus FVB/NJ-*Ugt1*^−/−^, at P2 and P5, respectively). Moreover, we observed that total plasma bilirubin of FVB/NJ-*Ugt1*^−/−^ mice significantly increased with time, reaching plasma bilirubin levels of C57BL/6-*Ugt1*^−/−^ levels in later time points (Fig. 1E; two-way ANOVA, interaction time-genotype, *P*≤0.0001; time, *P*≤0.0001, genotype, *P*≤0.0001; see also supplementary material Table S1).

To exclude the possibility that the differences in plasma TB and mortality could be associated with increased lysis of red blood cells in the C57BL/6 strain, we determined the erythrocyte osmotic fragility from both strains in WT P5 pups and adults. The neonatal period is known to have a high level of erythrocyte hemolysis (Pearson, 1967), but our data show that there were no differences between the strains in the NaCl concentration required for 50% hemolysis (C50), both in pups and adults (Fig. 1F,G, respectively).

### Bilirubin induces neurological damage in the cerebellum of FVB/NJ-*Ugt1*^−/−^ mutant mice

**Fig. 2.**
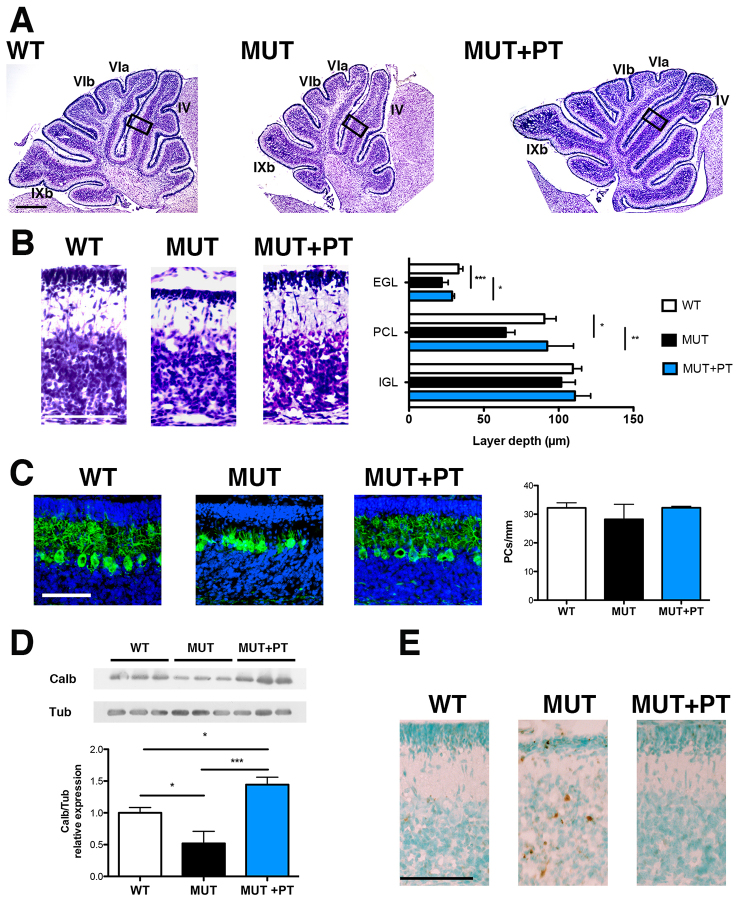
**Characterization of P8 cerebellar lesions in FVB/NJ-*Ugt1*^−/−^ mice with and without PT treatment.** (A) Nissl staining of 8-day-old (P8) WT, untreated mutant and PT-treated mutant mice. Boxed areas indicate fields shown in panel B. IV, VIa, VIb and IXb indicate the cerebellar fissures. Scale bar: 200 μm. (B) High-magnification images of cerebellar layers from P8 mice and, in the right panel, layer depth quantification (four mice/genotype). EGL, external germinal layer; PCL, Purkinje cell layer; IGL, internal granular layer. Scale bar: 100 μm. Error bars, s.d. (C) Representative fluorescent immunohistochemistry and quantification of PC number (cells/mm) from P8 WT, untreated mutant and PT-treated mutant mice. PCs were stained with anti-calbindin1 antibody (green) and nuclei with Hoechst staining (blue). Scale bar: 50 μm. Quantification of the PC number (cells/mm) is represented in the right panel (four mice/genotype). Error bars, s.d. One-way ANOVA test not significant. (D) Western blot analysis of total cerebellum protein extract using an anti-calbindin1 antibody in P8 WT, untreated mutant and PT-treated mutant mice. Tubulin was used as a loading control. Lower panel: densitometric quantification of the bands. (E) TUNEL analysis. Positive cells are shown as brown dots; negative cells are counterstained with methyl green. Scale bar: 100 μm. One-way ANOVA test. *P≤0.05; **P≤0.01; ***P≤0.001.

We exposed newborn mutant pups to blue light (λ=450 nm, 12 hour/day) from birth. Life-long PT treatment resulted in survival of all FVB/NJ-*Ugt1*^−/−^ mice, whereas all C57BL/6-*Ugt1*^−/−^ pups died within day 20 after birth (Fig. 1H). Because we have previously reported that bilirubin causes severe cerebellar abnormalities and death of C57BL/6-*Ugt1*^−/−^ mice, we analyzed brain morphology at P8 in untreated or PT-treated mutant mice and in WT littermates in FVB/NJ genetic background. We observed that untreated mutant cerebella showed important modifications of the architecture of the cerebellar fissures (Fig. 2A), in the depth of the cerebellar layers (Fig. 2B) and in the arborization of PCs (Fig. 2C). In contrast to what was observed in the C57BL/6-*Ugt1*^−/−^ strain, in which we reported a 37% reduction in PC number, we did not observe a significant decrease in PC density in cerebella of P8 FVB/NJ-*Ugt1*^−/−^ mice (Fig. 2C). We then performed a western blot analysis of cerebellar extracts using anti-calbindin antibody, a calcium-binding protein exclusively expressed by PCs in the cerebellum (Whitney et al., 2008), as a marker of PC impairment. Densitometric quantification of the bands revealed that untreated FVB/NJ-*Ugt1*^−/−^ mutant mice had a 50% reduction of calbindin content in their cerebella compared with WT littermates (Fig. 2D), confirming the observation of an important reduction in PC dendritic arbor. In addition, we performed terminal deoxynucleotidyl transferase-mediated deoxyuridine triphosphate nick-end labeling (TUNEL) analysis of entire brain sections and observed an increase of TUNEL-positive cells in the cerebellum of untreated mutant mice (Fig. 2E). TUNEL-positive cells were not restricted to a specific layer of the cerebellum: they were present in the external germinal layer (EGL), Purkinje cell layer (PCL) and in the internal granular layer (IGL). We analyzed other areas of the brain (cortex, hippocampus, basal ganglia), but we did not observe differences in their architecture, or an increase of TUNEL-positive cells, compared with WT littermates (data not shown).

PT treatment from birth fully rescued all features of bilirubin neurotoxicity (Fig. 2A–E).

### Bilirubin affects dendritic arborization and spine density of PCs *in vivo*

**Fig. 3.**
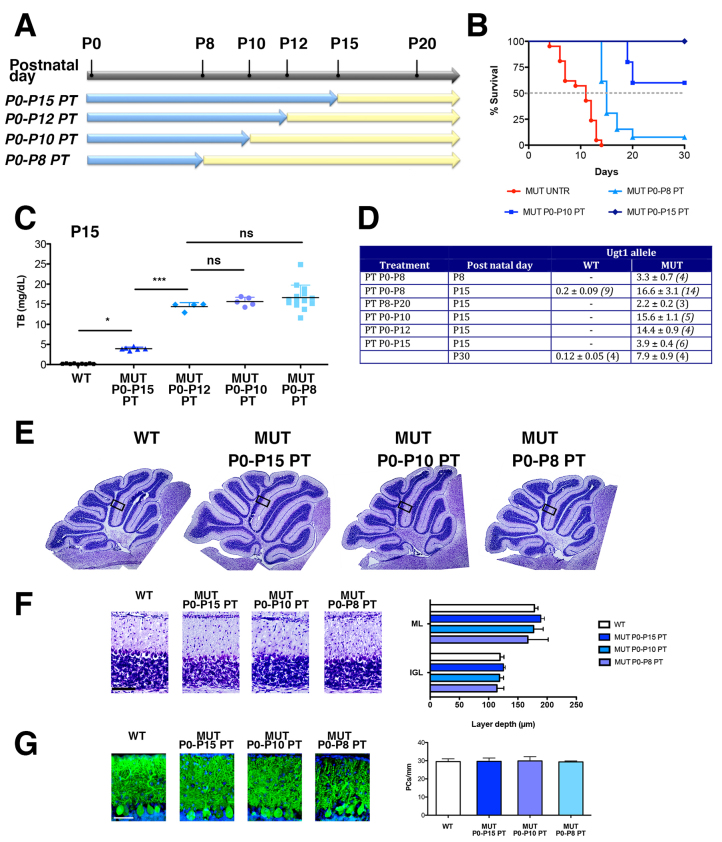
**Efficacy of PT treatment in FVB/NJ-*Ugt1*^−/−^ mice.** (A) Schematic representation of PT treatment. FVB/NJ-*Ugt1*^−/−^ mice and their WT littermates were exposed to PT (blue arrows) for different periods, starting at birth (P0) up to P8, P10, P12 and P15. At the indicated time points, mice were removed from PT and kept under normal light (yellow arrows). (B) Kaplan-Meier survival curves of FVB/NJ-*Ugt1*^−/−^ mice under normal light conditions (red line with ●, *n*=21), treated with PT for 8 days (light blue line with ▲, *n*=13), 10 days (blue line with ■, *n*=10) and 15 days (dark blue line with ♦, *n*=11) (*P*≤0.001). (C) Total plasma bilirubin (TB) levels at P15 of P0–P8, P0–P10, P0–P12 and P0–P15 PT-treated FVB/NJ-*Ugt1*^−/−^ mutant mice (*n*=14, 5, 4 and 6, respectively) and WT littermates (*n*=9). One-way ANOVA test with Bonferroni’s post-hoc comparison tests; ns, not significant; *P≤0.05, ***P≤0.001. (D) Total plasma bilirubin levels in WT and FVB/NJ-*Ugt1*^−/−^ mice. Values represent means±s.d. Values in parenthesis indicate number of samples analyzed. (E) Nissl staining of 15-day-old (P15) WT and PT-treated mutant mice. Boxed areas indicate fields shown in panel F. (F) High-magnification images of cerebellar layers from P15 mice and, in the right panel, layer depth quantification (four mice/treatment group). Scale bar: 100 μm. Error bars, s.d. One-way ANOVA test not significant. (G) Representative fluorescent immunohistochemistry and quantification of PC number (cells/mm) from P15 WT and PT-treated mutant mice. PCs were stained with anti-calbindin1 antibody (green) and nuclei with Hoechst staining (blue). Scale bar: 50 μm. Quantification of the PC number (cells/mm) is represented in the right panel (four mice/treatment group). Error bars, s.d. One-way ANOVA test with Bonferroni’s post-hoc comparison tests, not significant.

Next, we exposed FVB/NJ-*Ugt1*^−/−^ mice and their WT littermates to PT for different periods, starting at birth (P0), up to P8, P10 and P15. At the indicated time points mice were removed from PT and kept under normal light (Fig. 3A). As expected, survival of mutant mice was directly related to the extent of PT treatment (Fig. 3B). Discontinuation of PT treatment resulted in a rapid increase of bilirubin levels, reaching a plateau value of 14–16 mg/dl 3 days after PT removal (Fig. 3C,D), whereas, during PT treatment, mutant mice had TB levels of 3.9±0.4 mg/dl (Fig. 3D), well below those conferring the risk of developing neurological damage (Bortolussi et al., 2012). We analyzed cerebellar morphology at P15 and did not observe significant alterations of cerebellar fissures after the different treatments (Fig. 3E). Moreover, at this stage, cerebellar layer thickness was comparable among all treatments and was not statistically different from WT littermates (Fig. 3F). In addition, the PC number and calbindin content (quantified by densitometric analysis of the western blot bands) was comparable among all treatments and was not statistically different from WT littermates (Fig. 3G and supplementary material Fig. S1). These results indicated that PT treatment for 8 days after birth was sufficient to avoid major cerebellar abnormalities. However, calbindin staining of cerebella showed an apparent impairment of the PC dendritic arbor in P0–P8 PT-treated mutant mice, when determined at P15 (Fig. 3G).

**Fig. 4.**
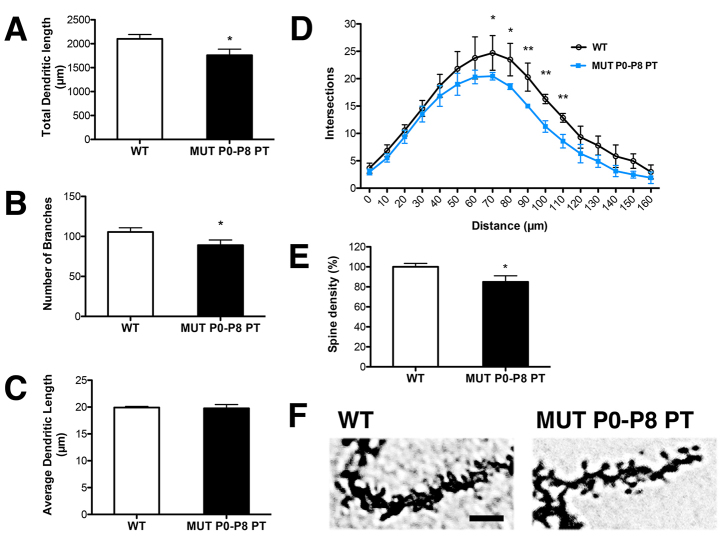
**Bilirubin reduces dendrite complexity and spine density of PCs.** Comparison of PCs from WT and P0–P8 PT-treated FVB/NJ-*Ugt1*^−/−^ mice at P15 (four mice/genotype). (A) Total dendritic length (μm). *t*-test analysis. *P≤0.05. (B) Number of branches. *t*-test analysis. *P≤0.05. (C) Average dendritic length (μm). *t*-test, not significant. (D) Sholl-analysis-derived distribution of PCs and mean number of intersections of dendrite branches. *t*-test analysis. *P≤0.05; **P≤0.01. (E,F) Spine density (%) and representative pictures of PC spines in WT and P0–P8 PT-treated mutant mice at P15. Mann-Whitney test analysis. *P≤0.01. Scale bar: 5 μm.

Owing to the high density and complexity of dendritic arbors in the PC layer, it was not possible to quantify the extent of PC impairment directly in anti-calbindin-immunostained sections. Thus, we assessed morphological changes in individual PCs by Golgi staining in P0–P8 PT-treated mutant mice at P15 and compared them with WT littermates (Fig. 4). Golgi staining is a very powerful technique that allows the analysis of morphological features such as dendritic arborization and spines of individual neurons in the brain (Ranjan and Mallick, 2010). After Golgi staining, ten single PCs with a complete dendritic arbor were selected from each animal (four animals/genotype). We reconstructed the dendritic arbor of each PC and measured three parameters: total dendritic length, number of branching points and average length of each branch. The total length of the PC dendritic arbor and the number of branching points from P0–P8 PT-treated mutant mice were reduced 16% and 17%, respectively, compared with WT littermates (Fig. 4A,B). In contrast, the average length of each branch (calculated as the ratio between total dendritic length and the number of branching points) was not reduced by bilirubin (Fig. 4C), suggesting that the entire dendritic arbor of PCs in P0–P8 PT-treated mutant mice was shorter in length owing to a reduction in the branching number, maintaining the same distance between successive branches. To more accurately assess subtle damage produced by bilirubin in P0–P8 PT-treated mutant mice, we performed the Sholl analysis of PCs, a quantitative method for morphometric neuronal studies (Sholl, 1953). Sholl analysis revealed that PCs from P0–P8 PT-treated mutant mice had a reduced dendritic complexity compared with WT controls. The reduction was particularly evident at a distance between 70 to 110 μm from the soma (Fig. 4D).

Moreover, to deeply characterize subtle bilirubin-induced damage, we measured spine density in PCs of P0–P8 PT-treated mutant mice and WT littermates. We observed that mutant mice had about 16% less spines/μm compared with healthy control animals (Fig. 4E,F).

In summary, these results show that the degree of bilirubin-dependent cerebellar neurodegeneration *in vivo* depends on the developmental stage of the specific cell type. Earlier bilirubin toxicity (P0–P8), as in untreated mutant animals, affects the granule cells (GCs) of the EGL and PCs, leading to decreased thickness of both the EGL and PCL, plus the almost total disruption of PC dendritic arbor. In contrast, later exposure (from P8 on) to high bilirubin levels affects only PC dendritic arborization and spine density. PCs receive impulses from several regions of the CNS, such as spinal cord, vestibular nucleus and cerebral cortex. Because their axons are the only synaptic output of the cerebellum, damage to these cells will deeply affect cerebellar functions.

### Phototherapy does not retrieve bilirubin-induced neurological damage accumulated up to P8

**Fig. 5.**
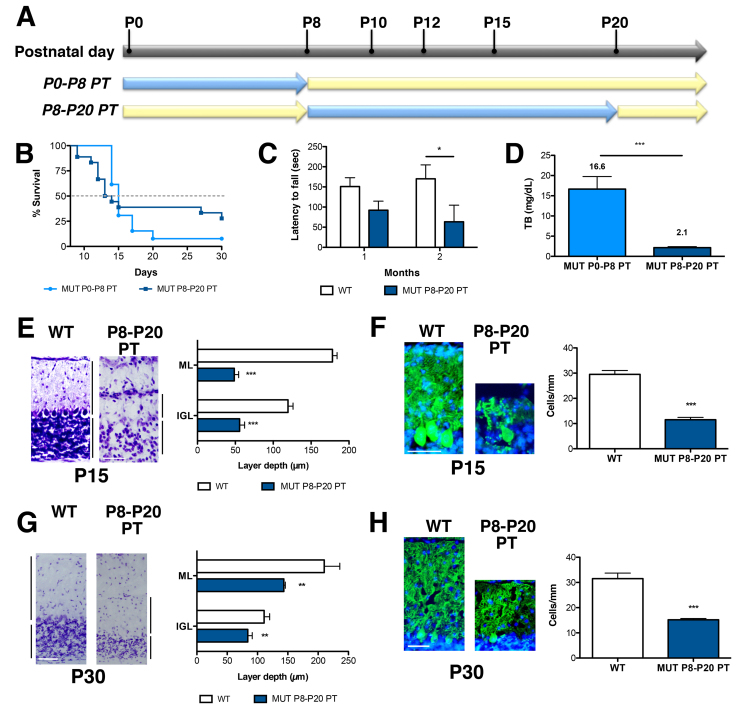
**Characterization of P15 cerebellar lesions in P8–P20 PT-treated FVB/NJ-*Ugt1*^−/−^ mice.** (A) Schematic representation of PT (blue arrows) treatment. (B) Kaplan-Meier survival curves of FVB/NJ-*Ugt1*^−/−^ mice treated with PT from P0 to P8 (light blue line with ●, *n*=13) and from P8 to P20 (dark blue line with ■, *n*=18). (C) Time course of motor coordination performance on rotarod of WT and P8–P20 PT-treated mutant mice (*n*=4/genotype). Two-way ANOVA test, interaction time-genotype, not significant (NS); time, NS; genotype, *P*≤0.01; Bonferroni’s post-hoc comparison tests: NS and *P*≤0.05 WT versus FVB/NJ-*Ugt1*^−/−^, at 1 and 2 months, respectively). *P≤0.01. WT, *Ugt1* wild-type allele; MUT, *Ugt1* mutant allele. (D) Total plasma bilirubin levels of P0–P8 and P8–P20 PT-treated mutant mice at P15 (*n*=14 and 4, respectively). *t*-test analysis. ***P≤0.0001. (E) High-magnification images of cerebellar layers from P15 mice and, in the right panel, layer depth quantification (four mice/genotype). ML, molecular layer; IGL, internal granular layer. Scale bar: 100 μm. Error bars=s.d. *t*-test analysis. ***P≤0.0001. (F) Representative fluorescent immunohistochemistry and quantification of PC number (cells/mm) from P15 mice. PCs were stained with anti-calbindin1 antibody (green) and nuclei with Hoechst staining (blue). Scale bar: 50 μm. Quantification of the PCs number (cells/mm) is represented in the right panel (four mice/genotype). Error bars=s.d. *t*-test analysis. ***P≤0.0001. (G) High-magnification images of cerebellar layers from P30 mice and, in the right panel, layer depth quantification (four mice/genotype). Scale bar: 100 μm. Error bars=s.d. *t*-test analysis. **P≤0.001. (H) Representative fluorescent immunohistochemistry and quantification of PC number (cells/mm) from P30 mice. PCs were stained with anti-calbindin1 antibody (green) and nuclei with Hoechst staining (blue). Scale bar: 50 μm. Quantification of the PC number (cells/mm) is represented in the right panel (four mice/genotype). Error bars=s.d. *t*-test analysis. ***P≤0.0001.

Because P8 seemed to be a key time point for bilirubin toxicity in the cerebellum of our FVB/NJ-*Ugt1*^−/−^ mice, next, we investigated whether the damage produced by bilirubin was reversible. Thus, mutant mice were left untreated up to P8, were then exposed to PT from P8 to P20 and their survival was monitored (Fig. 5A). Interestingly, we observed that we were able to rescue about 27% of mice receiving P8–P20 PT treatment (about 20% of mutant animals analyzed died before initiating the PT treatment and were not included in this calculation), whereas only 7% of the mutant animals treated from P0 to P8 with PT survived (Fig. 5B).

Survivors from P8–P20 PT treatment were tested for their motor coordination abilities on the accelerated rotating rod. The analysis was performed at 1 and 2 months of age (Fig. 5C). We observed that surviving mutant mice were severely impaired in their motor coordination performance compared with their WT littermates and that the difference was more evident at 2 months of age compared with 1 month (two-way ANOVA repeated measurements, Bonferroni’s post-hoc comparison test, 1 month *P*>0.05; 2 months *P*≤0.05, WT versus MUT P8–P20 PT).

We quantified TB levels at P15 and observed that PT treatment was very efficient in dropping down bilirubin levels from 16.6±3.1 mg/dl (TB levels of P0–P8 PT-treated mutant mice) to 2.1 mg/dl (Fig. 3D and Fig. 5D).

Histological analysis of the cerebellum of P8–P20 mutant mice highlighted an important cerebellar hypoplasia at P15. In fact, the molecular layer (ML) and IGL thickness was reduced about 73% and 54%, respectively, compared with WT littermates (Fig. 5E). We have described above that bilirubin decreases EGL and PCL thickness in untreated animals, but not IGL thickness, at P8 (Fig. 2B). We can speculate that the reduction seen in P8–P20 PT-treated mutant mice at P15 is the result of two main events: a stall in the duplication of GCs in the EGL, and cell death in all the layers of the cerebellum (see TUNEL analysis, Fig. 2E).

Interestingly, at the time when mutant animals were exposed to PT (at P8), they had high TB levels (12.5±0.9 mg/dl; Fig. 1E and supplementary material Table S1) and did not show an important reduction in PC number (Fig. 2C). However, after receiving 7 days of PT they showed low TB levels (Fig. 3D; PT P8–P20 group) but a 62% decrease in PC number (Fig. 5F), suggesting that exposure of PCs to bilirubin from P0 to P8 triggered substantial cell death events (see TUNEL analysis, Fig. 2E) that were not prevented by the successive PT treatment.

PT treatment was discontinued at P20 and 10 days later the cerebella of the mutant mice and their WT littermates were analyzed. ML and IGL thickness was still significantly reduced compared with WT littermates, although this difference was less pronounced than at P15 (by 32% and 24%, respectively; Fig. 5G). We speculate that the reduction of TB levels during P8–P20 allowed the spared GCs of the EGL to duplicate and recover part of the cerebellar architecture. The number of PCs decreased further; in fact there were 52% less compared with WT littermates (Fig. 5H). Thanks to PT treatment, the differences in layer thickness was reduced and spared PCs slightly improved their dendritic arbor compared with P8–P20 mutant animals analyzed at P15 (compare Fig. 5E,F to 5G,H).

### PT treatment for only 15 days is sufficient to fully rescue survival, and histological and behavioral features of FVB/NJ-*Ugt1*^−/−^ mice

**Fig. 6.**
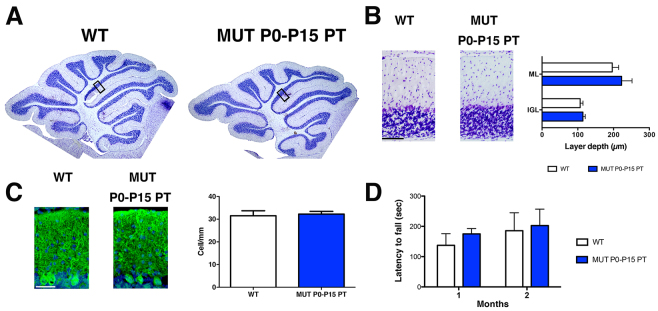
**Characterization of P30 cerebellar lesions in FVB/NJ-*Ugt1*^−/−^ mice exposed for 15 days to PT.** (A) Nissl staining of 30-day-old WT and P0–P15 PT-treated mutant mice. Boxed areas indicate fields shown in B. (B) High-magnification images of cerebellar layers from P30 mice and, in the right panel, layer depth quantification (four mice/genotype). Scale bar: 100 μm. Error bars, s.d. *t*-test analysis, not significant. ML, molecular layer; IGL, internal granular layer. (C) Representative fluorescent immunohistochemistry and quantification of PC number (cells/mm) from P15 mice. PCs were stained with anti-calbindin1 antibody (green) and nuclei with Hoechst staining (blue). Scale bar: 50 μm. Quantification of the PC number (cells/mm) is represented in the right panel (four mice/genotype). Error bars, s.d. *t*-test analysis, not significant. (D) Time course of motor coordination performance on rotarod of WT and P0–P15 PT-treated mutant mice at P30 and P60. Error bars=s.d. Two-way ANOVA repeated measurements, WT versus MUT P0–P15 PT, at P30 and P60, not significant.

When mutant mice were exposed to PT from P0 to P15, we observed 100% survival of treated mice (Fig. 3B). To determine whether 15 days of PT was sufficient to fully rescue mutant mice, we performed a more detailed histological, functional and molecular analysis. We observed that continuous PT treatment of mutant mice for 15 days prevented all morphological and functional deficits produced by bilirubin neurotoxicity, such as alterations in cerebellar morphology (Fig. 6A), and differences in layer thickness (Fig. 6B) and in the number and arborization of PCs (Fig. 6C). In addition, we observed that P0–P15 PT-treated mutant mice did not show any obvious motor coordination impairment as assessed by the accelerating rotarod test at any of the time points analyzed (Fig. 6D).

## DISCUSSION

Neonatal hyperbilirubinemia is the most common cause of hospital readmission in the first week of life (75%) (Escobar et al., 2005). There is a growing concern in the pediatric field regarding bilirubin neurotoxicity during the neonatal period because high bilirubin levels are life threatening and if neglected can lead to permanent brain disabilities and ultimately death. Moreover, infants show a huge variability in the extent of neurological damage caused by similar UCB levels, limiting the capacity to assess risk threshold for neurotoxicity and common intervention guidelines (Shapiro, 2003; Watchko and Jeffrey Maisels, 2010; Wennberg et al., 2006). During the past decades numerous progresses in understanding the mechanism by which bilirubin causes neurological damage have been made (Watchko and Tiribelli, 2013). However, the molecular and cellular mechanisms of bilirubin toxicity are still poorly defined owing to limitations of the available animal and cellular models.

To study bilirubin toxicity *in vivo*, we generated a mutant mouse model of neonatal hyperbilirubinemia lacking Ugt1a1 enzyme activity (Bortolussi et al., 2012). Mutant mice develop severe hyperbilirubinemia as early as 36 hours after birth, severe neurological damage and early lethality, recapitulating all major features of severe neonatal jaundice and bilirubin-induced neurological damage (BIND).

### Strain-specific bilirubin susceptibility

We characterized the lethal effects of *Ugt1* deficiency in two mouse genetic backgrounds, C57BL/6 and FVB/NJ, showing important differences in phenotype severity between the strains. In fact, mutant pups in the C57BL/6 genetic background had a median survival of 5 days (Bortolussi et al., 2012), whereas those in the FVB/NJ genetic background survived longer, with a median survival of 11 days. The differences in severity between the strains seem to be associated with the increased plasma bilirubin values in the C57BL/6 mice but not to a difference in erythrocyte fragility. We have shown that this difference is not associated with increased hemolysis and bilirubin production in the C57BL/6 strain, despite this strain having higher red cell count (10.3×10^6^ versus 9.2×10^6^, for C57BL/6 and FVB/NJ, respectively; http://phenome.jax.org) and hematocrit values (50.2 and 43.4, for C57BL/6 and FVB/NJ, respectively; http://phenome.jax.org).

Other genetically engineered mouse models, such as EGF receptor, retinoblastoma-related p130, fibronectin and Tgfb1 knockout (KO) strains, also showed that differences in the genetic background cause considerable variations in the phenotype (Doetschman, 2009; George et al., 1997; LeCouter et al., 1998a; LeCouter et al., 1998b; Threadgill et al., 1995). These important differences in phenotype severity are not yet fully elucidated, but seem to be associated with the modulatory effects of modifier genes (Astrof et al., 2007; Doetschman, 2009). Here, we have shown that the less severe phenotype of FVB/NJ-*Ugt1*^−/−^ mice correlates with the reduced cerebellar morphological defects, as compared with C57BL/6-*Ugt1*^−/−^ mice. In fact, C57BL/6 mutant mice showed a significant reduction both in the number of PCs and depth of cerebellar layers, whereas the number of PCs in FVB/NJ-*Ugt1*^−/−^ mice was not affected and the differences in cerebellar layers were less pronounced. A more detailed analysis showed that, in the FVB model, PC dendritic arborization is affected, similarly to that observed in C57BL/6-*Ugt1*^−/−^ pups (Bortolussi et al., 2012).

Interestingly, the mouse models characterized in this study have the same genetic mutation present in the hyperbilirubinemic Gunn rat (Bortolussi et al., 2012; Iyanagi et al., 1989), but displayed a much stronger phenotype. In fact, all mutant mice died a few days after birth due to bilirubin toxicity. Over time, different colonies of Gunn rats were used to study bilirubin toxicity, with phenotypes ranging from complete survival of untreated animals, with cerebellar abnormalities and hearing impairment, but reaching adulthood and reproducing (Chowdhury et al., 1993), to partial lethality (Keino and Kashiwamata, 1989; Keino et al., 1985–1986). Early studies in the Gunn rats (Johnson et al., 1959) reported plasma bilirubin levels similar to those found in the FVB/NJ-*Ugt1*^−/−^ mouse strain, although mortality was low and difficult to interpret owing to intercurrent infections. A more severe phenotype of the Gunn rats was obtained by treatment, at different postnatal days, with sulfadimetoxine, showing histological abnormalities in the cerebellar layers and PCs (Conlee and Shapiro, 1997; Schutta and Johnson, 1967; Schutta and Johnson, 1969; Schutta and Johnson, 1971) similar to those observed in our untreated mutant mouse strains. Other important differences are also observed when *Ugt1* mutant mice and the Gunn rats are exposed to PT: a single 24-hour dose of PT is enough to rescue Gunn rat lethality and prevent hypoplasia in the cerebellum, with treatment at day 7 being most effective (Keino and Kashiwamata, 1989) and the critical period for bilirubin-induced cerebellar hypoplasia being between P6 and P10 (Conlee and Shapiro, 1997; Keino and Kashiwamata, 1989; Sawasaki et al., 1976). In contrast, continuous PT is required in FVB/NJ-*Ugt1*^−/−^ mice to rescue lethality, whereas this treatment is not sufficient to prevent death of C57BL/6 mutant animals.

### Modulation of phenotype severity

We have presented here a novel and versatile model to study bilirubin toxicity. One of the advantages resides in the possibility to modulate phenotype severity by applying PT for different periods, allowing the study of physiological and biochemical implications of bilirubin toxicity at the desired developmental stage. For example, we studied the effects of bilirubin toxicity in mutant FVB/NJ-*Ugt1*^−/−^ mice treated with PT for 8 days. In these mice, despite the absence of gross cerebellar abnormalities, and a similar number of PCs and calbindin levels compared with WT mice, almost 90% of mutant mice did not survive. A more detailed analysis showed a significant reduction in dendritic arborization and in spine density, suggesting that these defects could contribute to neuronal abnormalities and death. When we extended PT treatment for another 2 days (P0–P10 PT), we observed a less severe condition, which resulted in about 50% survival.

We anticipate that these experimental conditions are valuable when the effect of a specific gene under study or pharmacological treatment is masked by the severity of hyperbilirubinemia, a condition that underestimates the therapeutic outcome or molecular partner contribution. Again, the readout of the experiment will be as simple as the increase of the survival rate.

### The window of neuronal susceptibility

It is known that the cerebellum is particularly vulnerable to insults because of its very rapid growth during peri- and postnatal development (Biran et al., 2012; Fonnum and Lock, 2000). Upon accomplishment of the early cerebellar patterning in which two germinal compartments (dorsal rhombic lip and the ventricular zone of the fourth ventricle) are generated, the cerebellar development experiences two main phases: (1) migration and proliferation of rhombotic lip progenitors to populate the external germinal layer; and (2) migration and differentiation of the PCs to populate the PCL (Roussel and Hatten, 2011).

Shortly after birth, GC progenitors undergo a strong and prolonged phase of clonal expansion in the EGL that lasts up to P20. The proliferation and migration of GCs is regulated by PC growth factors. After several cycles of duplication, GCs located in the external part of the EGL (facing the PCL) stop duplicating and migrate radially inward to their final destination in the IGL. During their migration into the IGL, GCs extend their T-shaped axons orthogonal to their migration path to generate the parallel fibers. Once in the IGL they will end their maturation process and receive input from the mossy fibers of the cortico-pontocerebellar neurons, the main input of the cerebellum. Concomitant with the onset of GC parallel fibers, PCs differentiate by completing their dendritogenesis.

**Fig. 7.**
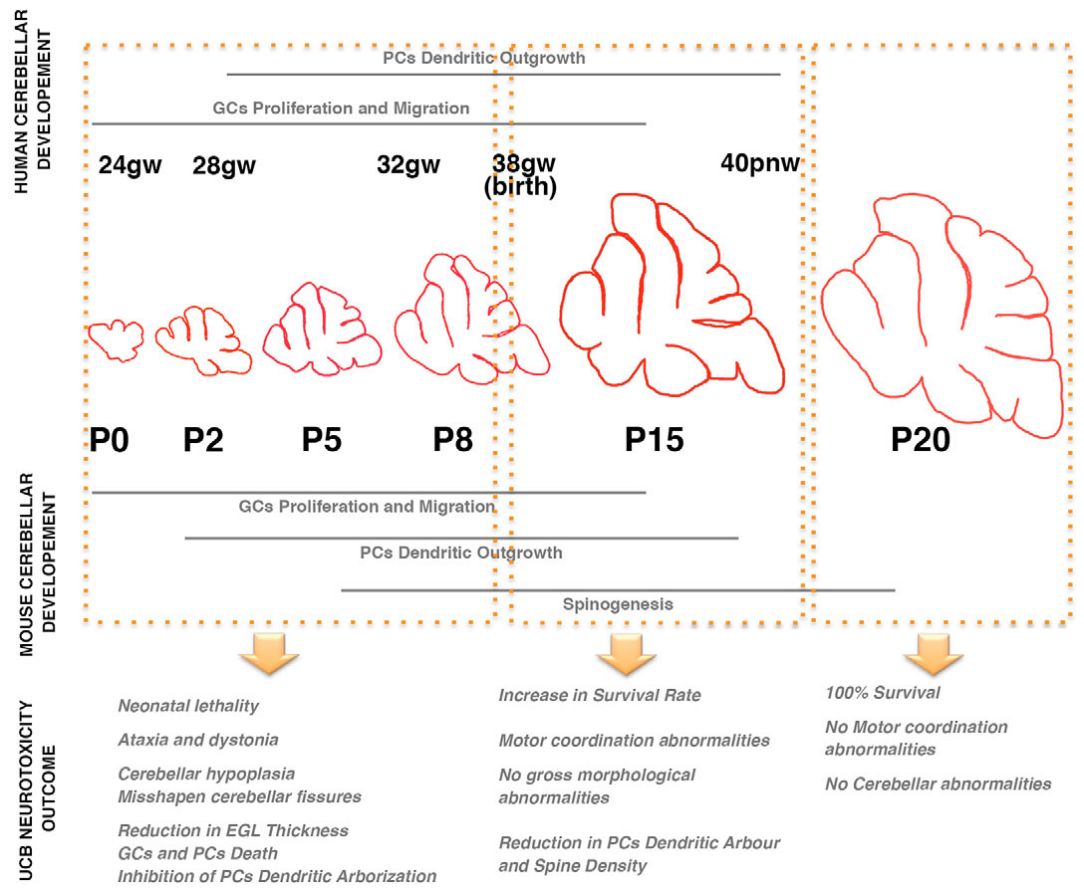
**Model of bilirubin toxicity during postnatal cerebellar development in the FVB/NJ-*Ugt1*^−/−^ mouse model.** Bilirubin toxicity during the neonatal period produces different outcomes depending on the developmental period of exposure. The window of high toxicity is between P0 and P8. The main events occurring in the cerebellum during this period are granule cell (GC) proliferation and migration, and Purkinje cells (PCs) extend their dendritic arbor. Bilirubin causes early neonatal lethality and substantial cerebellar damage. Later exposures (after P8) increase the number of spared animals and produce subtle damages, such as reduction of PC dendritic arbor and spine density. After P15, bilirubin does not cause lethality and evident cerebellar abnormalities. The comparison with human cerebellar development revealed that the critical window of toxicity is between the 24th and 38th gestational week (gw), also referred as the very preterm and preterm period, respectively. It is known that very preterm and preterm infants have a higher risk of developing bilirubin-induced neurological damage (BIND). The risk of developing BIND is critically reduced in term infant. pnw, postnatal week. Modified from Biran et al. (Biran et al., 2012).

Rodents and humans share a high degree of conservation of cerebellar anatomy and function, although the timing is slightly different (Biran et al., 2012). As described above, in rodents the proliferation of the EGL and the formation of the IGL occur postnatally, whereas the same phase in humans occurs between the 24th gestational week and birth (Fig. 7). Similarly, the main part of PC differentiation happens prenatally in humans, but postnatally in rodents. Thus, it is not surprising to note that cerebellar abnormalities in babies are a common complication of very premature and premature births (24–28 and 28–32 gestational weeks, respectively). Survivors frequently show a broad range of disabilities connected to cerebellar development, such as hypotonia, fine motor coordination failures, ataxia, impaired motor coordination sequencing and cerebral palsy (Haldipur et al., 2011; Limperopoulos et al., 2005; Volpe, 2009).

Preterm gestation is one of the most prevalent known risk factors for the development of severe hyperbilirubinemia and kernicterus (for a detailed review, see Watchko, 2006b); in fact, hyperbilirubinemia in preterm infants is more frequent and severe and its course is more prolonged then in term infants (Bhutani and Johnson, 2006). Treatment guidelines indicate that infants at higher risk to develop bilirubin-induced neurological sequelae are those born between the 35th and 37th gestational weeks (Watchko, 2006a). The spectrum of bilirubin-induced neurological disabilities also include movement disorders such as dystonia, athetosis, cerebral palsy and occasionally spasticity, which are well-known markers of basal ganglia, cerebellum and brainstem damage (Shapiro et al., 2006).

The results obtained in our mouse model mirror what has been reported so far in preterm and term infants. In fact, exposure of mutant mice to high levels of bilirubin during the first 8 days after birth (corresponding to preterm period in humans) causes severe neurological damage and a high mortality rate; at later time points (after P8, corresponding to term and late term infants), we reported a gradual decrease in the mortality rate, accompanied by less severe neurological outcomes (Fig. 7). It is well known that bilirubin neurotoxic effects can still be reversible if bilirubin levels are promptly reduced, avoiding permanent damage (Hansen et al., 2009; Zuccoli et al., 2011), although a range of outcomes suggests inter-individual variations in vulnerability (Hankø et al., 2001). In patients, the likelihood of reversing advanced stages depends on the implementation of rapid and effective treatments, such as intensive PT (Hansen, 2011) and exchange transfusion (Keenan et al., 1985; Roussel and Hatten, 2011).

In our FVB/NJ-*Ugt1*^−/−^ mice, the reversibility threshold seems to be before P8: when PT was applied from P8 and mutant mice analyzed at P15 (Fig. 5), a compromised motor-coordination function was observed, together with reduced depth of cerebellar layers, PC number and dendritic arborization. These damages were not reversed in spite of low plasma bilirubin values from P8 to P20 as the result of PT treatment. Such a critical period suggests that, in preterm infants, the identification and treatment of hyperbilirubinemia must be prompt and very efficient.

To summarize, we have shown here the consequences of the *Ugt1* null mutation in two mouse genetic backgrounds, with C57BL/6-*Ugt1*^−/−^ mice showing a more severe phenotype than FVB/NJ-*Ugt1*^−/−^ ones. We showed that PT treatment was effective in the prevention of brain damage and resulted in the complete rescue of the lethal phenotype only in the FVB/NJ-*Ugt1*^−/−^ animals. It emerges that susceptibility to bilirubin damage and, consequently, survival of mutant animals seems to be strain- and species-specific, underscoring the importance of modifier genes in the modulation of bilirubin toxicity.

The better characterization of the molecular mechanisms of bilirubin toxicity in the two mouse strains could result in the identification of novel genes involved in the modulation of bilirubin-induced neurological damage. Moreover, the capability to manipulate the mouse genome will allow the generation of mouse models of genes potentially involved in bilirubin metabolism that are expected to improve the comprehension of the mechanisms of UCB toxicity and protection. In view of these observations, FVB/NJ-*Ugt1*^−/−^ mice represent an excellent and versatile model to study bilirubin toxicity, the role of modifier genes and the effectiveness of new therapeutic approaches.

## MATERIALS AND METHODS

### Animals

*Ugt1* mutant mice in the C57BL/6 background have been generated previously (Bortolussi et al., 2012). WT littermates were used as a control. Mice were housed and handled according to institutional guidelines, and experimental procedures approved by the International Centre for Genetic Engineering and Biotechnology (ICGEB) board, with full respect to the EU Directive 2010/63/EU for animal experimentation. Animals used in this study were at least 99.8% C57BL/6 or FVB/NJ genetic background, obtained after more than nine backcrosses with C57BL/6 and FVB/NJ mice, respectively. Mice were kept in a temperature-controlled environment with 12/12 hours light/dark cycle. They received a standard chow diet and water *ad libitum*.

### Phototherapy treatment

Newborns were exposed to blue fluorescent light (20 μW/cm^2^/nm, Philips TL 20W/52 lamps; Philips, Amsterdam, The Netherlands) for 12 hours/day (synchronized with the light period of the light/dark cycle) up to the indicated postnatal day and then maintained under normal light conditions. Intensity of the blue lamps was monitored monthly with an Olympic Mark II Bili-Meter (Olympic Medical, Port Angeles, WA). The distance from the lamps to the bottom of the cases was ~27 cm.

### Bilirubin measurements

Blood samples were collected at different time points in mutant and WT littermates by decapitation or cardiac puncture in EDTA-collecting tubes. Total bilirubin (TB) determination in plasma was performed using Direct and Total Bilirubin Reagent kit (BQ Kits, San Diego, CA) adapting the method to use minimal volumes (10 μl of plasma). The adaptation of the method consisted in the reduction of the volumes (all), in order to use less sample volume. The original proportions were maintained. Three commercial bilirubin reference standards (Control Serum I, Control Serum II and Bilirubin Calibrator, Diazyme Laboratories, Poway, CA) were included in each set of analysis as quality control. Absorbance values at 560 nm were obtained by using a multiplate reader (Perkin Elmer Envision Plate Reader, Walthman, MA).

### Erythrocyte osmotic fragility test

The erythrocyte osmotic fragility (OF) test was performed as previously described (Muro et al., 2000) from freshly drawn blood. Five WT C57BL/6 and FVB/NJ animals per gender and time point were analyzed. The experiment was repeated three times with similar values. Results are expressed as percentage of lysis in graded salt concentrations (mean±s.d. of five males and five females). C_50_ values were determined by logarithmic linearization of the OF curve.

### Rotarod analysis

The coordination and balance ability on a rotating cylinder was assessed at 1 and 2 months of age with an accelerating apparatus as previously described (Bortolussi et al., 2012).

### Cerebellar histology

Brains were removed from the skulls and divided into two hemispheres: one was subjected to brain histology, whereas the other was subjected to protein extraction.

Brains from each genotype were fixed with 4% PFA in PBS overnight at 4°C. After cryoprotection in 20% sucrose, 0.02% sodium azide in PBS, specimens were frozen in cryostat embedding medium (Bio-optica) and 14-μm sagittal sections were obtained in a cryostat. Nissl staining was performed as previously described (Bortolussi et al., 2012). For immunofluorescence, 14-μm sagittal sections were blocked for 2 hours at room temperature (RT) with 2.5% BSA in PBS 0.3% Triton X-100. After blocking, specimens were incubated with the primary antibody for 2 hours at RT in blocking solution with anti-calbindin (Synaptic Systems, Goettingen, Germany). After 3×5-minute washes with blocking solution, specimens were incubated with secondary antibody (Alexa Fluor 488; Invitrogen Carlsbad, CA) for 2 hours at RT. Nuclei were visualized by addition of Hoechst (10 μg/ml, Invitrogen) for 5 minutes after secondary antibody solution.

TUNEL assay was performed using the *In Situ* Cell Death Detection Kit, POD according to manufacturer’s instructions (Roche). Methyl green was used as counterstaining.

Nissl-stained and TUNEL slides were mounted in Eukitt (Fluka, St Louis, MO), whereas immunostained slides were mounted in Mowiol 4–88 (Sigma-Aldrich).

Images were acquired on a Nikon Eclipse E-800 epifluorescent microscope with a charge-coupled device camera (DMX 1200F; Nikon Amstelveen, The Netherlands). Digital images were collected using ACT-1 (Nikon) software.

Analysis of the layer thickness was performed on Nissl-stained sections by measuring the layer depth (μm) as previously described (Bortolussi et al., 2012). PC number was calculated by counting calbindin-positive cells in vermis sections along the entire cerebellum perimeter and expressed as linear density (cell/mm) as previously described (Bortolussi et al., 2012).

Golgi staining was performed as described by Ranjan and Mallick (Ranjan and Mallick, 2010) with minor modifications to adapt the method to stain PCs of 15-day-old mice. Brains (*n*=4 mice for each group) were divided into the two hemispheres along the midline and, after a wash in PBS, they were submerged in the Golgi solution containing 5% of potassium dichromate, 5% of mercuric chloride and 5% of potassium chromate. Specimens were kept in darkness for 72 hours at 37°C. Golgi solution was replaced every day to increase the staining. Later, brains were washed in distillated water and sliced in a vibratome (Campden Instruments, MA752 motorised advance vibroslice) in 200-μm sagittal sections. Sections were then treated according to the Ranjan and Mallick protocol, dehydrated and mounted in Eukitt.

Ten PCs for each genotype/treatment were analyzed and a series of stack images were collected at 40× magnification. Stacks were flattened with ImageJ software to allow the quantification of specific parameters. Total dendritic length, number of branches, average of dendrite length and Sholl analysis were quantified using NeuroStudio software (Icahn School of Medicine at Mount Sinai, NY). Sholl analysis was performed with concentric circles of 10 μm. The complexity of the dentritic arbor is directly related to the number of times the dendrites cross the concentric circles (Sholl, 1953).

For spine density, ten segments of 50-μm long apical dendrites were analyzed for each animal (four animals/treatment) as previously described (Baj et al., 2014).

The genotype of the animals and the treatment were unknown to the operator. A second operator analyzed the data. Measurements were averaged for each animal. The results are expressed as mean±s.d. for each genotype.

### Preparation of total protein extracts and western blot analysis

Cerebella were dissected and homogenized in RIPA buffer (150 mM NaCl, 1% NP-40, 0.5% DOC, 0.1% SDS, 50 mM Tris HCl pH 8, 2× protease inhibitors) and analyzed by western blot analysis as described previously (Bortolussi et al., 2012). Primary antibodies used were as follows: anti-calbindin (Synaptic Systems, Goettingen, Germany) and anti-β-tubulin mAb E7 (Developmental Studies Hybridoma Bank, Iowa City, IA).

### Statistics

Results are expressed as mean±s.d. The Prism package (GraphPad Software, La Jolla, CA) was used to analyze the data. Values of *P*≤0.05 were considered statistically significant. Depending on the experimental design, we used Student’s *t*-test, one-way ANOVA or two-way ANOVA, with Bonferroni’s post-hoc comparison tests, as indicated in the legends to the figures and text.

## Supplementary Material

Supplementary Material
