# Supplementary material for: Age-dependent pattern of cerebellar susceptibility to bilirubin neurotoxicity in vivo in mice
Source: Dis Model Mech. 2014 Jul 25;7(9):1057–68. doi: 10.1242/dmm.016535 (PMC4142726; doi:10.1242/dmm.016535)
Supplement: Supplementary Material [file supp_7_9_1057__index.html]

Age-dependent pattern of cerebellar susceptibility to bilirubin neurotoxicity in vivo in mice — Supplementary Material 

# Age-dependent pattern of cerebellar susceptibility to bilirubin neurotoxicity *in vivo* in mice

## DMM016535 Supplementary Material

**Files in this Data Supplement:**

- **Supplementary Material**
