# Supplementary material for: Age-dependent pattern of cerebellar susceptibility to bilirubin neurotoxicity in vivo in mice
Source: Dis Model Mech. 2014 Jul 25;7(9):1057–68. doi: 10.1242/dmm.016535 (PMC4142726; doi:10.1242/dmm.016535)
Supplement: Supplementary Material [file supp_7.9.1057_DMM016535.pdf]

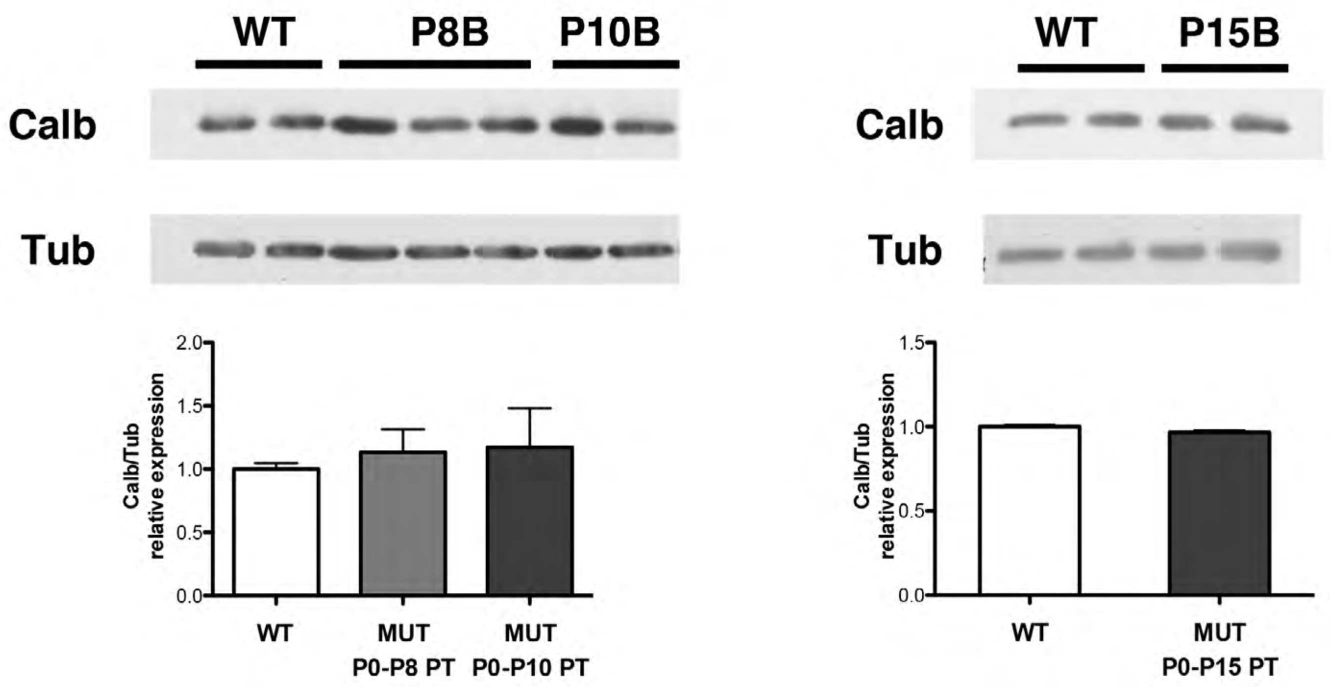

Supplementary Fig. 1. Western blot analysis of total cerebellum protein extracts using an anti-calbindin1 antibody in P15 WT and PT-treated mutant mice. Tubulin was used as a loading control. Lower panel: densitometric quantification of the bands. ML, molecular layer; IGL, internal granular layer.

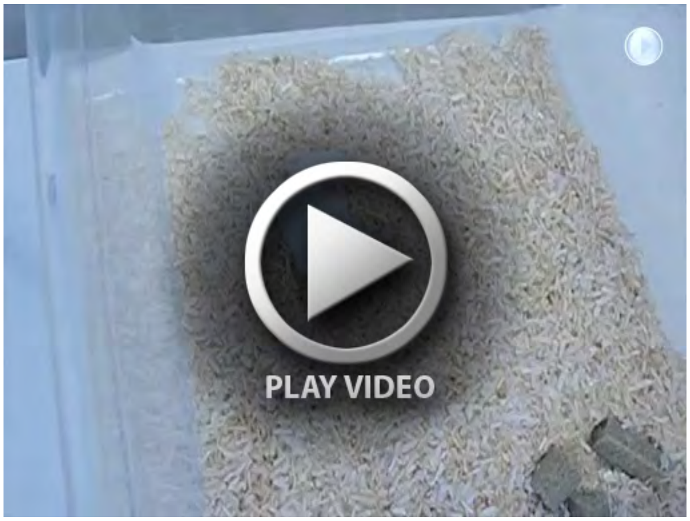

Movie 1. Untreated FVB/NJ-Ugt1$^{-/-}$ at P12 showing features of bilirubin encephalopathy such as lethargy, dystonia and seizures.

Genetic Background	Post natal day	Ugt1 allele<br>WT	 MUT
C57Bl/6	P2	0.2 ± 0.1 (8)	11.8 ± 2.2 *(6)*
	P5	0.2 ± 0.1 (9)	12.9 ± 1.7 (10)
FVB/NJ	P2	0.3 ± 0.09 *(5)*	*8.8 ± 0.9 (3)*
	P5	0.2 ± 0.02 *(4)*	10.4 ± 0.5 *(8)*
	P8	0.2 ± 0.08 *(7)*	12.5 ± 0.9 *(8)*
	P10	0.2 ± 0.1 *(5)*	14.4 ± 0.8 *(3)*

Supplementary Table 1. Total plasma bilirubin levels in C57Bl/6 & FVB/NJ mice. Values represent means±SD. Values in parenthesis indicate number of samples analysed.
